# A novel stream of collaborative National portals to enhance preparedness and informed choices

**DOI:** 10.1093/eurpub/ckac129.642

**Published:** 2022-10-25

**Authors:** F Carinci, C Vicario, N Zamperini, F La Valle, L Rocchi, G Veltro, G Siccardi, D Mantoan

**Affiliations:** 1 Information and Communication Technologies, Italian National Agency for Regional Health Services, Rome, Italy; 2 Department of Statistical Sciences, University of Bologna, Bologna, Italy; 3 Gnoti Lab srl, Rome, Italy

## Abstract

**Issue/problem:**

AGENAS supports the implementation of health policies in direct collaboration with Italian Regions and Autonomous Provinces. To improve public reporting, we aimed to complement the production of technical reports with new forms of timely communication, using National Portals.

**Description of the problem:**

Between October-December 2020, we designed and implemented the Covid-19 National Portal, including a suite of targeted indicators, fully automated via ad hoc scripts written in php and R on top of a relational database using internal and external data sources. Targeted information was widely communicated and continuously updated. Dedicated sections on forecasting and resilience were delivered in collaboration with specialised academic institutions. In 2021, we deployed the Portal for the Transparency of Health Services, broadly oriented towards health issues, the location of services and performance indicators.

**Results:**

Pre-post comparisons of web analytics for Jan-Apr 2020-2022 showed clear advantages of Covid-19 Portal. By Apr 2020, Italy had introduced national lockdown, while AGENAS covered the topic traditionally, recording 48,122 users overall, with daily peaks below 5,000 sessions. In 2021-2022, the number of users skyrocketed at 436,280, with daily peaks of 100,000 sessions, and 421,123 respectively, with daily peaks of 150,000 sessions. Visits to the Transparency Portal were considerably more limited.

**Lessons:**

To be widely used, public health information needs to be relevant (responding to personal need close to home), understandable, accurate and timely. National Portals can gain efficiency through the mediation of search engines, enhanced by: targeted naming (url), coherent semantic perimeter (third level domain in a highly referenced institutional website), continuous updating, and impact factor (linked by authoritative websites). The Transparency Portal will take stock of these lessons to succeed in a new funded program of NextGenerationEU.

**Key messages:**

• Relevant, understandable, accurate and timely dissemination for different types of audience may be effectively organised through National Portals.

• Productive collaboration between health specialists and communication experts can enhance usability and actionability of National Portals for public health.

